# The Impact of H1–H4 Receptor Antagonists on the Levels of Selected Oxidative Stress Markers in Liver and Muscle Tissue in an Animal Model of Colitis

**DOI:** 10.3390/ph19010177

**Published:** 2026-01-20

**Authors:** Bartosz Bogielski, Katarzyna Michalczyk, Wojciech Gębski, Katarzyna Rozpędek, Elżbieta Szulińska, Bartosz Tempka, Aleksandra Zorychta, Elżbieta Chełmecka, Ewa Kaczmar, Piotr Głodek, Jakub John, Kamil Nikiel, Bronisława Skrzep-Poloczek, Jerzy Jochem, Katarzyna Kieć-Kononowicz, Dorota Łażewska, Dominika Stygar

**Affiliations:** 1 Branch in Bielsko-Biała, Medical University of Silesia, 43-382 Bielsko-Biała, Poland; bartosz.bogielski@sum.edu.pl; 2 Department of Physiology, Faculty of Medical Sciences in Zabrze, Medical University of Silesia, 41-800 Zabrze, Polands80900@365.sum.edu.pl (P.G.); bskrzep-poloczek@sum.edu.pl (B.S.-P.); jjochem@sum.edu.pl (J.J.); dstygar@sum.edu.pl (D.S.); 3 Institute of Biology, Biotechnology and Environmental Protection, Faculty of Natural Sciences, University of Silesia in Katowice, 40-007 Katowice, Poland; katarzyna.rozpedek@us.edu.pl (K.R.); elzbieta.szulinska@us.edu.pl (E.S.); 4 Department of Medical Statistic, Faculty of Pharmaceutical Sciences in Sosnowiec, Medical University of Silesia, 41-200 Sosnowiec, Polandechelmecka@sum.edu.pl (E.C.); 5 Department of Clinical Diagnostics, Faculty of Veterinary Medicine, University of Warmia and Mazury in Olsztyn, 10-719 Olsztyn, Poland; ewa.kaczmar@uwm.edu.pl; 6 Department of Technology and Biotechnology of Drugs, Faculty of Pharmacy, Jagiellonian University Medical College, 30-688 Kraków, Poland; katarzyna.kiec-kononowicz@uj.edu.pl (K.K.-K.);

**Keywords:** colitis, histamine receptor antagonists, oxidative stress markers

## Abstract

**Background/Objectives:** The global prevalence and incidence of inflammatory bowel diseases have risen in the past two decades. Among them, Crohn’s disease and ulcerative colitis are still challenging to treat due to vascular and proliferative alterations. Studies in rats suggest that blocking histamine receptors (H1–H4) can improve colitis progression. However, the specific histamine receptor responsible for this effect remains debated. The experiment aimed to assess the role of specific histamine receptor subtypes in colitis development, focusing on oxidative stress markers in the liver and skeletal muscle. **Methods:** The study involved 60 adult male Wistar rats, divided into control and colitis experimental groups. Colitis was induced through intracolonic administration of 2,4,6-trinitrobenzenesulfonic acid. Animals in both experimental groups received intramuscular injections of NaCl (non-treated, NT) or H1, H2, H3, and H4 receptor antagonists (10 study subgroups in total). On day eight, the animals were re-anesthetized and euthanized via exsanguination. Then, liver and skeletal muscle (*m. soleus*) samples were collected for analysis of oxidative stress markers. **Results:** The analyses of skeletal muscle samples showed that using the H1 and H2 receptor antagonists increased superoxide dismutase (SOD) and catalase (CAT) activities, as well as parameters related to glutathione metabolism (reduced glutathione (GSH), glutathione S-transferase (GST)) in rats from the control groups, indicating enhanced antioxidant defense. In rats with chemically induced colitis, we observed that H1 receptor antagonists elevated CAT activity, whereas β-esterase (β-EST) activity remained elevated across all colitis subgroups. In the liver, histamine receptor antagonists produced receptor-specific redox effects: the H2 receptor antagonist reduced oxidative damage (malondialdehyde (MDA)); the H1 receptor antagonist attenuated SOD hyperactivity, but depleted GSH; and the H4 receptor antagonist increased GSH while elevating MDA. Chemically induced colitis increased α- and β-EST activities, whereas administration of the H1 or H3 antagonist reduced β-EST levels. **Conclusions:** Histamine receptor antagonists modulated oxidative stress responses in both liver and skeletal muscle tissues in a receptor-dependent manner. Among them, the H2 receptor antagonist most effectively mitigated hepatic oxidative injury, highlighting its potential as a therapeutic target in colitis-associated systemic oxidative stress.

## 1. Introduction

Crohn’s disease and ulcerative colitis, the inflammatory bowel diseases (IBD) that have become more prevalent worldwide over the past decades and gained status as civilization diseases [1], continue to pose significant therapeutic challenges in clinical practice [2,3,4,5,6,7] due to vascular and proliferative alterations that play a crucial role in the pathogenesis of both of these disease entities. Histamine, a key mediator in the mammalian gastrointestinal tract, not only stimulates gastric acid secretion or modulates motility in the stomach and intestines, but also regulates blood flow through the gastrointestinal vasculature [8,9,10]. Studies in various rodent experimental models suggest that blocking histamine reduces colitis progression. However, there is ongoing debate over the specific histamine receptor(s) responsible for this effect [11,12,13,14,15,16,17,18,19]. Fogel et al. [12] reported that histamine contributes to colonic inflammation in a rat model of experimental ulcerative colitis, suggesting a role for histaminergic mechanisms in disease pathophysiology; however, this early work did not delineate specific receptor subtypes. In a related study, Fogel et al. [18] investigated the influence of the H3/H4 receptor antagonist, thioperamide, on regional hemodynamics in rats with the 2,4,6-trinitrobenzenesulfonic acid (TNBS)-induced colitis, suggesting that pharmacological modulation of these receptors can alter inflammatory responses and local circulatory dynamics in the inflamed gut, albeit without isolating the distinct contributions of H3 vs. H4 receptors [18]. In contrast, evidence from more recent receptor-specific investigations, such as those examining the effects of histamine H4-receptor deletion in mice, indicates that H4 signaling may mediate anti-inflammatory effects in experimental colitis models, with H4-deficient animals exhibiting more severe disease symptoms [11]. Collectively, these findings show that different histamine receptor subtypes may affect intestinal inflammation differently, and highlight the need for further studies to clarify which receptors are principally responsible for histamine’s effects in inflammatory bowel disease models.

In 1978, Guth & Smith showed that infusing histamine into the superior mesenteric artery resulted in a dose-dependent reduction in vascular resistance, leading to increased blood flow in this region. This effect was inhibited by the H1 receptor antagonist mepyramine, suggesting that H1 receptors mediate this process [20]. It has also been reported that histamine enhanced blood flow in the mesenteric circulation [21,22,23,24], while blocking histamine H3 and H4 receptors simultaneously decreased blood flow in the inferior mesenteric artery and inhibited the development of the inflammatory process [10]. Evidence from other studies suggests that blocking H3 receptors with clobenpropit reduced reactive hyperemia in the intestinal circulation of rats, indicating a role for H3 receptors in modulating blood flow following ischemic episodes [25].

Existing reports suggest that the regulatory mechanisms of the circulatory system in rats closely resemble those in humans. Current evidence underscores the translational utility of rat models in cardiovascular research, primarily due to conserved regulatory mechanisms in blood pressure control, neurohormonal signaling, and pharmacological responses [26,27,28,29,30,31]. Notably, oxidative stress pathways in vascular endothelial cells exhibit striking evolutionary conservation between rats and humans, reinforcing the relevance of rodent models for preclinical testing of anti-inflammatory therapies [32,33].

Additionally, the TNBS-induced colitis model in rats is a well-established model of non-specific intestinal inflammation in humans [34,35,36] because it reproduces key features of human inflammatory bowel disease, particularly transmural inflammation, Th1/Th17-driven immune responses, and disruption of the epithelial barrier, which resemble, for example, aspects of Crohn’s disease [37,38,39]. Moreover, this model is highly reproducible and allows precise control of disease onset and severity. On the other hand, the TNBS model does not fully recapitulate the chronic and relapsing nature of human IBD and may be influenced by chemical injury-related mechanisms. But in contrast, other models, such as dextran sulfate sodium (DSS)-induced colitis, primarily reflect epithelial barrier damage and innate immune activation, whereas genetic or spontaneous models better mimic chronic immune dysregulation [38,40]. Apart from that, studies in rodent models show that chronic intestinal inflammation accompanying TNBS- or DSS-induced colitis triggers a systemic surge of reactive oxygen species (ROS) and proinflammatory cytokines, which disrupts homeostasis in peripheral tissues [41,42,43,44,45,46,47,48,49], contributing to extra-intestinal organ dysfunction, especially in the liver [48]. Several studies have demonstrated that chemically induced colitis leads to significant oxidative damage in hepatic tissue, indicating a strong gut–liver axis mediated by oxidative mechanisms. Increased ROS generation, lipid peroxidation, and antioxidant depletion in hepatic tissue have been documented in IBD models and patients [40,41,42,43,44,45,46,47,48,49,50,51,52]. Furthermore, clinical and experimental reviews show that oxidative and nitrosative stress exacerbate intestinal mucosal injury and contribute to extraintestinal manifestations, such as hepatobiliary complications [53].

Laroui et al. [41], studying a rodent model of TNBS- or DSS-induced colitis, showed that chronic intestinal inflammation triggers a systemic surge of ROS and proinflammatory cytokines (e.g., TNF-α, IL-6, IL-1β), which disrupt redox homeostasis in peripheral tissues, particularly the skeletal muscles. Accumulating evidence underlines a complex interplay between colitis-associated inflammation, systemic oxidative stress, and skeletal muscle dysfunction driven by multifactorial pathways.

Current knowledge on the role of individual histamine receptors in the healing process, particularly through the analysis of selected biochemical parameters in colitis models, remains limited. The presented study aimed to assess the role of specific histamine receptor types in the development of colitis in rats. It also explored the link between the role of histamine receptors in relaying the systemic oxidative stress accompanying the ulcerative colitis and its impact on peripheral tissues such as liver and skeletal muscles.

## 2. Results

### 2.1. Oxidative Stress Markers in the Liver Tissue

When comparing the effects of the histamine receptor antagonists, we found differences in SOD activity between the colitis control and colitis groups for all treatment groups except for rats that were treated with the histamine H4 receptor antagonist (*p* = 0.575, Table 1).

The analysis showed statistically significant differences in SOD activity in the liver of rats from the colitis control (*p* < 0.001) and from the colitis groups (*p* < 0.001). Among the colitis control groups, a difference was found between non-treated and histamine H2 receptor antagonist-treated rats (*p* < 0.05). Among the colitis groups, we found differences in SOD activity between non-treated and treated rats, with SOD activity lower in the treated groups, except in those treated with the histamine H2 receptor antagonist (Table 2).

Similarly to SOD, CAT activity in the liver of rats also differed between the colitis control (*p* < 0.001) and colitis groups (*p* < 0.001). Detailed analysis showed that CAT activity was higher in histamine H1 (*p* < 0.01) and H2 (*p* < 0.001) receptor antagonist-treated group than in the non-treated group of rats from the colitis control groups, but in the colitis groups was higher in the non-treated groups of rats than in rats treated with histamine H3 (*p* < 0.01) and H4 (*p* < 0.001) receptor antagonists (Table 2).

When comparing treatment effects, we found that histamine receptor antagonist treatment significantly lowered CAT activity in the colitis groups regardless of the targeted receptor type (Table 1). Also, we noted that in the non-treated groups of rats, CAT activity was higher in rats with chemically induced colitis.

The activity of GST differed significantly between the colitis control and colitis groups (*p* < 0.001 for both). However, detailed analysis showed that within the colitis control group GST activity was significantly different in the liver of rats treated with histamine H1 receptor antagonist (*p* < 0.01), while within the colitis group it was different in the liver of rats treated with histamine H2 (*p* < 0.001) and H4 (*p* < 0.01) receptor antagonist (Table 2) when compared to the respective non-treated group.

When comparing the treatment effect, we found that GST activity in the liver of rats with colitis was significantly different from its activity in the liver of rats from the respective colitis control group, both in treated and non-treated rats (Table 1).

The analysis of the results showed that GSH concentration differed significantly between the colitis control (*p* < 0.001) and colitis groups (*p* < 0.001) (Table 2). Further analysis within the groups showed that GSH concentration in the colitis control groups was significantly different only between the non-treated rats and the rats that were treated with the histamine H1 receptor antagonist (*p* < 0.01). Within the colitis groups, the non-treated rats presented higher GSH concentration than rats treated with histamine H2 (*p* < 0.001) or H3 receptor antagonists (*p* < 0.01) (Table 1 and Table 2).

When comparing the colitis control and the colitis groups from different treatment groups, we found that GSH concentration was significantly lower in rats treated with histamine H1 and H2 receptor antagonists (*p* < 0.01 for both) and followed that trend in rats from the colitis groups treated with the histamine H3 receptor antagonist (Table 1). GSH concentration in these rats treated with a histamine H4 receptor antagonist was higher than in the liver of rats from the respective colitis control group (*p* < 0.01) (Table 1).

Initial analysis showed statistically significant differences in MDA concentration within the colitis control and colitis groups (*p* < 0.01 for both comparisons). Further analysis within the colitis control groups showed no differences in MDA concentration in the liver of rats from the non-treated and treated groups (Table 2). The analysis within the colitis group showed that MDA concentration was significantly higher in the liver of rats treated with the histamine H4 receptor antagonist than in the liver of the non-treated rats (*p* < 0.05) (Table 1 and Table 2).

When comparing the effects of the treatment between the colitis control and colitis groups, we found that MDA concentration was higher in the liver of rats treated with histamine H2 (*p* < 0.01) and H3 receptor antagonist (*p* < 0.01) when compared to the respective colitis controls (Table 1). The analysis showed statistically significant differences in TAC levels in the liver tissue of rats from the colitis control (*p* < 0.001) and the colitis groups (*p* < 0.001) (Table 2). More detailed analysis showed that among the colitis control groups, the difference occurred between rats from the non-treated and rats treated with the histamine H3 receptor antagonist (*p* < 0.001), while among the colitis groups, the difference occurred between the non-treated colitis group and rats treated with histamine H2 (*p* < 0.01), H3 (*p* < 0.001), and H4 (*p* < 0.05) receptor antagonist (Table 2).

When comparing the effect of specific histamine receptor antagonists on TAC levels, we found the differences between the colitis control and colitis groups for the non-treated rats (*p* < 0.01), and rats treated with the histamine H2 (*p* < 0.01) and H4 (*p* < 0.05) receptor antagonist (Table 1).

The analysis indicated that TOS levels in the liver tissue were significantly different within the colitis control (*p* < 0.01) and the colitis groups (*p* < 0.05). However, more detailed analysis showed no differences between TOS levels of the non-treated and any of the histamine receptor antagonist-treated rats from both groups (Table 2).

When comparing the effects of histamine receptor antagonists, we found differences in TOS levels in the liver of non-treated (*p* < 0.01) and histamine H1 receptor antagonist-treated (*p* < 0.01) rats between the colitis control and the colitis groups (Table 1). In both cases, TOS levels in the liver of rats from the colitis control group were higher than those from the colitis group.

Analysis of EST activities showed that α-EST activity in the liver was the same across all colitis control groups (Table 2). In the colitis groups, the analysis showed differences within the groups (*p* < 0.01): α-EST activity in the liver of rats treated with the histamine H4 receptor antagonist was significantly different from α-EST activity in the non-treated group (*p* < 0.05) (Table 2).

When comparing the effects of the treatment, we found that α-EST activity was lower in the liver of rats treated with the histamine H1 receptor antagonist and in the liver of the non-treated rat from the colitis group compared to the respective colitis control group (*p* < 0.01 for both) (Table 1).

As for β-EST activity, the analysis showed a statistically significant difference between the colitis control and colitis groups (*p* < 0.001 for both). Detailed analysis within the colitis control groups did not confirm any differences between β-EST activity in the liver of non-treated rats and rats that received the treatment (Table 2). In the colitis groups, we found that treating the rats with histamine H1 or H3 receptor antagonist resulted in lower β-EST activity when compared to the non-treated group (*p* < 0.05 for the H1 group and *p* < 0.001 for the H3 group) (Table 1 and Table 2).

We found that β-EST activity in the non-treated group of rats with chemically induced colitis was significantly higher than in the non-treated rats from the colitis control group (*p* < 0.01). We found that β-EST activity significantly decreased in the liver of rats from the colitis groups when comparing the effect of the histamine receptor antagonist between the respective colitis control and the colitis groups.

### 2.2. Oxidative Stress Markers in the Muscle Tissue

We observed differences in superoxide dismutase (SOD) activity in the non-treated (*p* < 0.05), H1 (*p* < 0.01), and H2 receptor antagonist-treated (*p* < 0.01) rats. We noted higher SOD activity in muscle tissue from non-treated colitis rats. In contrast, the H1 and H2 receptor antagonist-treated groups showed higher SOD activity in the muscle tissue sampled from the colitis control groups. When comparing SOD activity for each group separately, we found differences in SOD activity between the non-treated rats and the H1 receptor antagonist-treated rats (*p* < 0.05) and H2 receptor antagonist-treated rats (*p* < 0.05) within the colitis control groups. Higher SOD activities were observed in the muscle tissues sampled from rats treated with histamine receptor antagonists. We noted no differences in SOD activity within the colitis groups.

Analysis of catalase (CAT) activity in the colitis control and colitis groups showed that it differed in the non-treated (*p* < 0.01), H1 (*p* < 0.01), and H4 receptor antagonist-treated (*p* < 0.05) groups. Higher CAT activities were observed in the muscle tissue sampled from rats with chemically induced colitis of the non-treated and the H4 receptor antagonist-treated rats, while it was lower in the muscle tissue samples from the H1 receptor antagonist-treated rats (Table 3). When analyzing each group separately, we found that within the colitis control groups, the H1 and H2 receptor antagonists-treated rats presented higher CAT activity than the non-treated rats (*p* < 0.01 in both cases). Within the colitis groups, higher Cat activity was noted only in the H1 receptor antagonist-treated rats compared to the non-treated rats (*p* < 0.01) (Table 4).

We observed differences in glutathione S-transferase (GST) activity in the muscle tissue samples between the colitis control and the colitis groups of rats treated with H1 and H2 receptor antagonists, with the colitis groups showing higher GST activities (Table 3). When comparing GST activity within each colitis group separately, we found no statistical differences (*p* = 0.268). However, within the colitis control groups, we found that the GST activity was significantly higher in the muscle tissue of H1 receptor antagonist-treated rats than in the NT rats (*p* < 0.001) (Table 4).

As for the GSH concentration, the analysis of the results showed differences between the colitis control and colitis groups only in the case of the non-treated and H1 and H2 receptor antagonist-treated rats. Higher GSH concentrations were observed in the non-treated rats from the colitis group (*p* < 0.01), whereas within the colitis control groups, higher GSH concentrations were observed in the muscle tissue of the H1 and H2 receptor antagonist-treated rats (in both cases, *p* < 0.01) (Table 3). No differences in GSH concentrations were observed within the colitis groups, whereas within the colitis control groups, treating the rats with H1 or H2 receptor antagonists significantly increased GSH concentrations in the muscle tissue (*p* < 0.01 in both cases) (Table 4).

Regarding malondialdehyde (MDA) concentration, we observed statistical differences only in the H1 receptor antagonist-treated group (Table 3), in which colitis was associated with a lower MDA concentration. However, adding any of the other antagonists did not result in differences in MDA between colitis and non-colitis rats. Considering only the effects of the histamine receptor antagonists within the colitis control and colitis groups, the analysis indicated differences between the histamine receptor antagonists, but more detailed comparisons of concentration did not confirm that (Table 4).

Analysis of the results showed the differences in total antioxidant capacity (TAC) values in the soleus muscle samples between the colitis control and colitis groups for the non-treated (*p* < 0.01), H1 (*p* < 0.05), and H3 (*p* < 0.001) receptor antagonist-treated rats. We noted lower TAC values in the colitis groups, except for the non-treated rats. Comparing TAC values within the colitis control and colitis groups revealed statistically significant differences in both groups (*p* < 0.001 and *p* < 0.01, respectively). However, detailed analyses did not indicate differences within the colitis control group. Within the colitis groups, we observed differences in the TAC values between all treated groups and the non-treated group, except for the H2 receptor antagonist-treated rats.

Comparing the effects of specific receptor antagonists between the colitis control and colitis groups, we found differences in total oxidative status (TOS) values in the soleus muscle sampled from all groups: non-treated and treated with histamine receptor antagonists. We noted higher TOS values in the colitis control groups compared to the colitis groups, except for the muscle tissue samples from the H3 and H4 receptor antagonist-treated groups (Table 3). Comparing TOS values within the colitis control and colitis groups, we found statistically significant differences within both groups (Table 4). Detailed analyses showed no differences in the effects of various antagonists within the colitis group, whereas in the colitis control groups, we observed differences when comparing the non-treated with H1 (*p* < 0.01) or H3 receptor antagonist group (*p* < 0.05) (Table 2).

Comparing the effects of specific histamine receptor antagonists, we noted higher α-esterase (EST) activities in the soleus muscle of rats from the colitis control group than the colitis group for the non-treated rats (*p* < 0.05) and rats treated with the H1 receptor antagonist (*p* < 0.01). The initial analysis of the α-EST activity indicated statistically significant differences within the colitis control (*p* < 0.05) and in the colitis groups (*p* < 0.01). However, detailed analyses revealed no differences in α-EST activity in the soleus muscle samples collected from the non-treated rats and rats treated with either of the histamine antagonists (Table 4).

As for β-EST activity, comparing the effects of specific receptor antagonists revealed differences between the soleus muscle samples from the colitis control and colitis groups of the non-treated and all histamine receptor antagonist-treated rats, except for rats treated with the H3 receptor antagonist (Table 3). We found statistically significant differences in β-EST activity within the colitis control (*p* < 0.01) and the colitis group (*p* < 0.001). Detailed analyses of β-EST activity in the soleus muscle samples of the colitis control group showed differences between the non-treated and the H4 receptor antagonist-treated group (*p* < 0.01). In the colitis group, we found significant differences between the non-treated rats and rats treated with H1 (*p* < 0.05) or H4 (*p* < 0.001) antagonists (Table 4).

## 3. Discussion

Accumulating evidence underlines a complex interplay between colitis-associated inflammation and systemic oxidative stress driven by multifactorial pathways.

Histamine plays a multifaceted role in inflammatory redox biology by modulating ROS generation via different receptor-mediated pathways. Histamine can enhance ROS generation through H1 receptor activation—studies in neutrophils have demonstrated that high concentrations of histamine stimulate ROS production via H_1_R and the NADPH oxidase complex. Mast cells further illustrate histamine’s redox interplay; upon antigenic or neuropeptide stimulation, they generate intracellular ROS, which is associated with histamine release and may amplify inflammatory signaling [54]. Akamatsu et al. [54] investigated whether azelastine (0.05–5 μg·mL^−1^), a second-generation H1 blocker, could modulate oxidative burst in human neutrophils. The results confirmed significant inhibition of superoxide anion, hydrogen peroxide, and hydroxyl radical production [55]. It has been shown that histamine triggers neutrophil extracellular trap (NET) formation through NADPH oxidase-dependent mechanisms involving ERK and p38 pathways [56].

The histamine receptor antagonists used in this study are well-established compounds with documented subtype selectivity. Cetirizine is a highly selective second-generation H1 receptor antagonist with negligible activity towards H2, H3, and H4 receptors at pharmacologically relevant concentrations, while ranitidine is a selective H2 receptor antagonist with minimal cross-reactivity toward other histamine receptor subtypes [57,58,59]. Iodophenpropit is a potent H3 receptor antagonist/inverse agonist with high affinity for H3 receptors, but it shows limited interaction with the H4 receptor due to structural homology between these receptors [60,61]. Finally, JNJ7777120 is widely used as a selective H4 receptor antagonist, but studies indicate potential partial agonist activity under certain experimental conditions, which should be considered when interpreting H4-mediated effects [62,63].

### 3.1. Oxidative Stress in the Liver of Rats with Chemically Induced Colitis: Effects of Treatment with Histamine Receptor Antagonists

Emerging evidence suggests that histamine signaling may contribute to the pathogenesis of immune-mediated liver injury, offering potential therapeutic targets. The liver expresses all four histamine receptor subtypes (H_1_R-H_4_R), with H_1_R and H_4_R appearing particularly relevant to inflammatory processes [64,65,66,67,68]. Administration of histamine receptor antagonists has shown promise in a range of hepatopathies. For instance, combined H1/H2 blockade with mepyramine and ranitidine in Mdr2^−^/^−^ mice—an established model of primary sclerosing cholangitis (PSC) and cholangiocarcinoma—resulted in significantly reduced biliary damage, hepatic fibrosis, cholangiocyte proliferation, angiogenesis, and epithelial–mesenchymal transition (EMT) compared to controls [65]. This supports the potential of H_1_/H_2_ antagonists in ameliorating PSC-associated liver injury. In immunized rabbit models, pheniramine (H_1_ antagonist) and ranitidine (H2 antagonist) significantly altered serum ALT, AST, ALP, and bilirubin levels, indicating a modulatory effect on hepatic inflammation and function during immune challenge [65]. Furthermore, cimetidine has been shown to inhibit hepatic microsomal drug-metabolizing enzymes in a dose-dependent fashion, suggesting H_2_-mediated regulation of phase I detoxification pathways [69]. In a rat ischemia–reperfusion model, activation of H_4_R via agonists such as dimaprit and clozapine markedly reduced ALT and AST levels, an effect reversed by the H3/H4 antagonist thioperamide—highlighting a protective, H_4_R-mediated mechanism during ischemic stress [70]. Similarly, in an LPS/GalN murine model of acute hepatic inflammation, oral administration of H_4_R antagonists (JNJ-7777120, JNJ-28307474) attenuated Kupffer cell-derived TNF-α expression in the liver and lowered ALT concentrations, thereby alleviating liver injury [71].

The obtained data demonstrate that selective blockade of histamine receptor subtypes (H1–H4) exerts distinct effects on liver oxidative stress parameters in a TNBS-induced rat colitis model, suggesting potential extra-intestinal hepatic involvement during intestinal inflammation. However, to our knowledge, this is the first report demonstrating a consistent effect of H1–H4 receptor antagonists decreasing CAT activity in colitic liver tissue, extending previous findings on receptor-specific oxidative stress modulation.

Given that the liver is the primary organ for detoxification and metabolic regulation, the observed alterations in antioxidant enzymes and oxidative markers may reflect secondary hepatic inflammatory responses to colitis or direct effects of histamine receptor modulation on hepatocyte function.

We found that H1 receptor blockade with cetirizine (H1 receptor antagonist) significantly altered hepatic oxidative stress markers in TNBS-induced colitis, suggesting a potential protective effect against colitis-associated liver damage. Additionally, the antagonist of H2, H3, and H4 receptors significantly elevated hepatic TAC levels in colitis groups, with the H3 antagonist (iodophenpropit) showing the most pronounced effect. This suggests that H3 receptors may play a unique role in liver redox homeostasis, possibly by modulating Kupffer cell activation or hepatocyte antioxidant gene expression. The colitis-induced hepatic SOD hyperactivity, attenuated by H1 and H2 antagonists, but exacerbated by H3 receptor blockade, could indicate either compensatory antioxidant upregulation due to systemic inflammation or direct histaminergic regulation of hepatic superoxide metabolism. The suppression of CAT activity by the H3 or H4 antagonist in colitis rats may impair H_2_O_2_ clearance in hepatocytes, potentially exacerbating oxidative liver injury during intestinal inflammation.

The elevated MDA levels in colitis groups further support the notion of colitis-associated hepatic oxidative stress, possibly due to increased gut-derived pro-inflammatory mediators (e.g., TNF-α, LPS) reaching the liver via the portal circulation. The fact that the H2 receptor antagonist (ranitidine) partially reversed these effects suggests that histamine H2 receptors may contribute to hepatic oxidative damage during colitis, possibly by influencing sinusoidal blood flow or hepatocyte mitochondrial function. However, the unexpected pro-oxidant effect of the H4 antagonist (increased MDA) raises concerns about its potential hepatotoxicity, despite its beneficial elevation of hepatic GSH. This dual effect warrants further investigation into whether H4 receptor blockade alters hepatic lipid peroxidation pathways or impairs mitochondrial function, especially since Schirmer et al. [72], using a dual colitis-hepatitis model, proved that blocking H_4_R with JNJ7777120 reduced portal inflammation and IL-6 levels by 62% compared to controls, suggesting gut-liver axis modulation.

The depletion of hepatic GSH by the H1 and H2 antagonists could reflect either increased utilization due to inflammation or direct inhibition of glutathione synthesis in hepatocytes. In contrast, the H4 antagonist’s ability to elevate hepatic GSH suggests a possible hepatoprotective mechanism, potentially via Nrf2 pathway activation. The altered GST activity further supports histamine receptor-dependent modulation of hepatic detoxification capacity, which may influence drug metabolism during colitis. The reduction in hepatic α- and β-esterase activities in H1- and H3-receptor antagonist-treated rats suggests that histamine receptors may regulate phase I hepatic metabolism during inflammation, possibly affecting the clearance of xenobiotics or endogenous toxins.

Scientific reports have highlighted potential hepatotoxic effects associated with histamine H2 receptor antagonists. Liver injury induced by these drugs is relatively rare but clinically significant, characterized predominantly by cholestatic or mixed hepatocellular-cholestatic patterns. It is currently hypothesized that two primary mechanisms underlie this hepatotoxicity: metabolic disturbances associated with cytochrome P450 enzyme inhibition and immunologically mediated hypersensitivity reactions. Specifically, drugs such as cimetidine and ranitidine can inhibit hepatic CYP450 enzymes, disrupting metabolic pathways within hepatocytes [73].

### 3.2. Oxidative Stress in the Skeletal Muscles of Rats with Chemically Induced Colitis: Effects of Treatment with Histamine Receptor Antagonists

Laroui et al. [41], studying a rodent model of TNBS- or DSS-induced colitis, showed that chronic intestinal inflammation triggers a systemic surge of reactive oxygen species and proinflammatory cytokines (e.g., TNF-α, IL-6, IL-1β), which disrupt redox homeostasis in peripheral tissues, particularly the skeletal muscles. Similarly, Sui et al. [42] found that gut bacteria exacerbate TNBS-induced colitis and kidney injury through oxidative stress, further disrupting redox balance. Ranganathan et al. [43] reported that blocking CXCR2 receptors mitigates DSS-induced inflammation and oxidative stress, protecting peripheral organs. Additionally, Hambardikar & Mandlik [46] demonstrated that naringin improves antioxidant status and reduces proinflammatory cytokines in TNBS-induced colitis, indicating a protective effect on redox homeostasis in peripheral tissues. Our findings align with this paradigm, as we observed a significant reduction in TAC in the soleus muscle of rats with TNBS-induced colitis, especially in H1- and H3-antagonist-treated groups. We observed highly consistent modulation of TOS in the soleus muscle, with all comparisons reaching *p* < 0.01 significance—a uniformity that underscores histamine’s non-redundant role in oxidative regulation. Specifically, control animals maintained systematically higher TOS than colitis groups across all treatments except for H3 and H4 antagonists, where this relationship inverted. This reversal may indicate a distinct role for H3 and H4 receptors in modulating peripheral oxidative stress, potentially compensatory under inflammatory conditions. The consistent reduction in TOS in colitis models suggests a shift in redox homeostasis, possibly reflecting altered mitochondrial function or antioxidant responses in skeletal muscle under systemic inflammation [74,75]. Such findings are consistent with the hypothesis that chronic intestinal inflammation in IBD may contribute to secondary myopathies by dysregulating oxidative metabolism [76,77,78,79,80,81,82]. The study by Sieck et al. [83] provides evidence that histamine signaling influences skeletal muscle function, particularly in the context of endurance exercise adaptations. Additionally, the review by Smolinska et al. [9] suggests that histamine plays a role in the inflammatory processes associated with IBD, which could have implications for muscle function during systemic inflammation. While direct evidence linking histamine, IBD, and skeletal muscle dysfunction is limited, these studies suggest that histamine signaling may contribute to muscle dysfunction under inflammatory conditions [83,84,85].

Chronic inflammation, such as that induced by colitis, leads to significant alterations in oxidative stress markers and antioxidant enzyme activities in skeletal muscle. Studies have demonstrated reduced activity of key antioxidant enzymes, including SOD, CAT, and GST, alongside decreased GSH levels in muscle tissue [86,87]. For instance, Wei et al. [85] reported that elevated ROS levels activate nuclear factor erythroid 2-related factor 2 (Nrf2), which subsequently upregulates the expression of antioxidant enzymes, such as SOD, CAT, and GST in skeletal muscle.

The results obtained in the control group showed that cetirizine and ranitidine, H1 and H2 antagonists, respectively, demonstrated a consistent ability to increase the activity of key antioxidant enzymes (SOD, CAT) and glutathione-related parameters (GSH, GST), highlighting their potential in enhancing antioxidant defense in non-inflammatory conditions. The results obtained in the colitis groups suggest that H1 and H2 antagonists maintained some influence on antioxidant enzymes, particularly CAT activity, indicating partial preservation of antioxidant responses under inflammatory conditions. The obtained results show that SOD and CAT play a key role in neutralizing ROS in both physiological and pathological states. The consistent increase in their activities observed in H1- and H2-receptor antagonist-treated rats suggests that H1 and H2 receptors are integral to antioxidant regulation. The dual actions of H1 and H2 antagonists and their respective receptors in modulating oxidative stress suggest their potential as adjunct therapeutics for managing inflammatory conditions such as colitis.

Mitochondrial dysfunction emerges as a central mechanism, with colitis-induced ROS overproduction impairing electron transport chain (ETC) complexes I and III, reducing ATP synthesis, and activating proteolytic pathways (e.g., ubiquitin-proteasome system, UPS) that drive muscle atrophy [88]. Notably, NF-κB and MAPK signaling pathways are hyperactivated in myocytes during colitis, further amplifying inflammatory and catabolic responses [89,90]. Our findings reveal that histamine receptors critically influence this redox-mitochondrial axis, as demonstrated by the exacerbated oxidative stress in muscle tissue, evidenced by reduced TAC values after blocking H1 and H3 receptors.

Colitis-associated dysbiosis disrupts the gut-muscle axis and exacerbates systemic oxidative stress via bacterial metabolite shifts (e.g., increased lipopolysaccharide, LPS) and impaired short-chain fatty acid (SCFA) production, which is a critical modulator of muscle redox balance. Clinical studies showed that IBD patients exhibit elevated serum ROS levels and reduced muscle mass that correlate with disease severity [91,92,93,94,95]. The preclinical models of therapeutic interventions targeting oxidative stress, such as N-acetylcysteine (NAC), resveratrol, or mitochondrially targeted antioxidants (e.g., MitoQ), efficiently attenuated muscle atrophy and restored glutathione pools [96].

The altered α- and β-esterase activities in the soleus muscle suggest that colitis-induced systemic inflammation impairs enzymatic defense mechanisms in the skeletal muscles. The reduced α-EST activity in rats with colitis, particularly in the non-treated and the H1 antagonist-treated groups, may suggest that the detoxification pathways and macrophage function might be suppressed by inflammation [97,98]. β-EST activity showed a greater activity decrease, except for the H3 antagonist treated rats, supporting the idea of a possible protective role of H3 receptor blockade in preserving oxidative homeostasis [99]. Studies show that H3 receptors modulate oxidative stress and inflammation, and their antagonism has protective effects in ischemia and IBD models [100,101]. In contrast, blocking the H4 receptor exacerbated the reduction in both esterase activities. Given the immunoregulatory role of the H4 receptor, its blockade may disrupt immune balance and enhance oxidative damage in the muscles [102,103,104].

These enzymatic shifts may be early signs of IBD-associated myopathy, as skeletal muscle dysfunction is common in IBD and linked to oxidative stress, mitochondrial alterations, and systemic inflammation [105,106,107].

Our results suggest that glutathione metabolism appears more sensitive to inflammation. We observed elevated glutathione-related markers (GST activity, GSH concentration) in the soleus muscle of control rats treated with H1 and H2 antagonists and decreased values of these markers in the soleus muscle of rats with chemically induced colitis, effects that are probably due to overwhelming oxidative stress. Histamine H3 and H4 receptor antagonists exhibited minimal impact on oxidative stress parameters in rats with induced colitis. However, when analyzing the role of H3 antagonist in the control rats, we noted that it significantly reduced the concentration of MDA, reflecting the potential role of iodophenpropit and H3 receptors in protecting against lipid oxidation damage under non-inflammatory conditions. Malondialdehyde levels were not significantly modulated in rats with induced colitis, suggesting that inflammatory processes overshadow lipid-specific oxidative responses. In rats with chemically induced colitis, neither H3 nor H4 receptor antagonists significantly modulated oxidative stress markers in the soleus muscle, suggesting a more limited role for H3 and H4 receptors in this pathological model and a need for further investigation into their roles and mechanisms.

The presented study findings align with previous studies indicating the involvement of histamine receptors in inflammatory modulation and oxidative stress [108]. This suggests that H1 and H2 receptor antagonists may serve as potential adjunctive IBD therapies modulating oxidative stress and inflammation. However, H3 and H4 receptor antagonists did not significantly influence oxidative stress parameters in either the control or the colitis groups, which is consistent with earlier findings on the effects of iodophenpropit and JNJ7777120 in other inflammatory settings [109].

Although histamine receptor antagonists have been investigated in various inflammatory conditions, their potential therapeutic role in myopathies remains largely unexplored. While our findings indicate that H1 and H2 receptor antagonists influence oxidative stress markers, their direct effect on muscle function in IBD-associated sarcopenia requires further research. Current literature lacks studies assessing histamine receptor blockade as a therapeutic approach for IBD-related muscle atrophy. However, evidence suggests that chronic inflammation and oxidative stress play a central role in muscle degradation in colitis models [110].

### 3.3. Limitations of the Study

Despite analyzing the responses of oxidative stress markers in liver and skeletal muscle tissue to multiple histamine receptor antagonists, we should acknowledge several limitations of the study.

The TNBS-induced colitis is a well-established model. However, it primarily recapitulates acute transmural inflammation characteristic of Crohn’s disease rather than chronic ulcerative colitis [111]. This limits direct translational applicability to human IBD phenotypes, particularly those characterized by long-term mucosal healing and fibrosis. Additionally, the study exclusively used male Wistar rats, which makes it impossible to assess the sex-specific responses or estrogen-mediated gastroprotection observed in female IBD patients [111].

Additionally, the doses for H1–H4 receptor antagonists were selected only based on literature, without formal pharmacokinetic/pharmacodynamic optimization in Wistar rats with the chemically induced colitis. Therefore, we cannot exclude potential off-target effects, variable receptor occupancy, or altered pharmacokinetics due to colitis-induced gut dysfunction. Moreover, plasma histamine and histamine receptor expression levels were not measured to confirm target receptor engagement.

Finally, regarding methodology, the analyzed tissues were collected only on day 8 after colitis induction. It means that the presented results reflect health status at peak acute inflammation but miss the early initiation of the inflammatory cascade (days 1–3) and the chronic remodeling phases (>14 days). Dynamic longitudinal sampling would better delineate receptor-specific temporal redox responses.

As for the results, the concerns regarding ranitidine (H2 antagonist) hepatotoxicity [73] should be taken into account when interpreting the observed MDA reduction. On the other hand, JNJ7777120 (H4 antagonist) showed dual GSH↑/MDA↑ effects, which suggests potential hepatotoxicity that would require dose–response clarification.

Finally, Janus kinase (JAK) inhibitors also represent a clinically validated therapeutic strategy in inflammatory bowel disease, particularly ulcerative colitis. The pan-JAK inhibitor, tofacitinib, demonstrated efficacy in inducing and maintaining clinical and endoscopic remission in phase 3 OCTAVE trials, while the selective JAK1 inhibitor, upadacitinib, showed comparable efficacy in large randomized studies [112,113]. Preclinical studies in chemically induced colitis models, including TNBS-induced colitis, indicate that JAK inhibition attenuates mucosal inflammation, reduces myeloperoxidase activity, suppresses pro-inflammatory cytokines, and improves epithelial barrier function [40,114]. Given the bidirectional relationship between JAK/STAT signaling and oxidative stress—where reactive oxygen species activate STAT pathways and cytokine-driven JAK signaling promotes oxidative injury—JAK inhibition has been associated with improved redox homeostasis in inflammatory conditions [115,116]. In this context, our findings demonstrate that histamine receptor antagonism modulates oxidative stress markers in extraintestinal tissues in a receptor-dependent manner, suggesting a complementary regulatory axis influencing systemic oxidative stress during colitis.

These limitations highlight the need for chronic colitis models, multi-organ longitudinal assessments, pharmacodynamic confirmation, and functional/clinical correlations to fully validate histamine receptor modulation as IBD therapy.

## 4. Materials and Methods

### 4.1. Ethical Permissions

The study design and experimental procedures received approval from the Local Ethical Committee for Animal Experimentation at the Medical University of Silesia (Katowice, Poland) (Approval No. 66/2021, dated 15 December 2021). The research was conducted following institutional and national ethical guidelines for the care and use of animals, as outlined in Directive 2010/63/EU.

### 4.2. Study Object, Number of Animals, and Housing Conditions

The study used 60 adult, 3–4-month-old male Wistar rats (*Rattus norvegicus*) weighing 200 ± 20 g. The animals were purchased from the Center for Experimental Medicine of the Medical University of Silesia in Katowice, Poland.

The number of animals used in the study was minimized in compliance with the 3Rs principles of humane animal research, as proposed by Russell and Burch in The Principles of Humane Experimental Technique (1959) [117]. Group sizes were determined through a power analysis performed using Statistica 13 software (Statistica 13.0, StatSoft Polska, Kraków, Poland), ensuring that the minimum number of animals required for statistical reliability was used while maintaining appropriate significance levels for this type of research [118,119,120]. The analysis accounted for an expected effect size of 1.5, α = 0.05, and β = 0.2, as recommended for preclinical studies [120].

The rats were housed in groups of two individuals per cage. The cages (58.5 cm × 37.5 cm × 20 cm—floor area: 920 cm^2^) were equipped with environmental enrichment, including plastic shelters and wooden blocks.

Housing conditions were strictly controlled, maintaining a temperature of 20–22 °C, relative humidity of 50–60%, air exchange rate of 15/h, and a 12 h light/12 h dark cycle.

Rats underwent a 7-day acclimatization period before starting any experimental procedures.

### 4.3. Experimental Design

Following a 7-day acclimatization period, the animals were randomly assigned to 10 groups, each comprising six individuals. Five groups served as colitis controls to five experimental groups with chemically induced colitis. Colitis was induced via intracolonic administration of 2,4,6-trinitrobenzenesulfonic acid (TNBS, 25 mg/0.8 mL in 37% ethanol) using a flexible polyethylene catheter (0.8 mm diameter, 8 cm length). The colitis control groups received an equivalent volume of 0.9% NaCl solution. Both TNBS and NaCl were administered under general anesthesia, which was induced by intraperitoneal injection of ketamine (Bioketan, 115.34 mg/mL) at 100 mg/kg and xylazine (Sedazin, 20 mg/mL) at 10 mg/kg [121,122,123,124].

For seven consecutive days during the post-induction phase, rats from the colitis control and colitis groups received intramuscular injections of:

- 0.2 mL of 0.9% NaCl in the non-treated (NT) groups
- 10 mg/kg of cetirizine in 0.2 mL of 0.9% NaCl [107] in the H1 receptor antagonist-treated groups
- 10 mg/kg of ranitidine in 0.2 mL of 0.9% NaCl [123] in the H2 receptor antagonist-treated groups
- 10 mg/kg of iodophenpropit in 0.2 mL of 0.9% NaCl [108] in the H3 receptor antagonist-treated groups
- 30 mg/kg of JNJ7777120 hydrochloride in 0.2 mL of DMSO [124] in the H4 receptor antagonist-treated groups.

On the eighth day, the animals were re-anesthetized following the previously described protocol. Euthanasia was carried out by exsanguination via a cannula inserted into the right femoral artery. Blood and tissue samples were subsequently collected for biochemical, molecular, and histopathological analyses.

#### Dosing of Used Chemicals

The TNBS dose was selected because it is a documented and reproducible regimen in rat colitis models that yields robust, yet survivable, inflammation [125].

For anesthesia, the ketamine–xylazine (K/X) combination at 100/10 mg/kg i.p. was selected because it is a well-established protocol for rats, widely validated across experimental settings. For example, Wellington et al. [126] demonstrated stable anesthesia in Wistar rats with this regimen, and IACUC guidelines consistently list ketamine 40–100 mg/kg with xylazine 5–13 mg/kg as standard ranges. These doses are also routinely applied in gastrointestinal inflammation models, including TNBS-induced colitis [127]. Importantly, ketamine acts as an NMDA receptor antagonist and xylazine as an α2-adrenergic agonist, acting through non-histaminergic pathways [121]. This minimizes the likelihood of confounding effects on histamine receptor antagonists. Indeed, recent rodent studies employing JNJ7777120 and other histamine receptor blockers under K/X anesthesia have not reported any interference with antihistaminic activity [122,128].

The difference in H1-H4 antagonists dosing reflects receptor-specific pharmacodynamics and the available preclinical evidence supporting efficacy at different dose ranges. For H1–H3 antagonists, the literature supports effective dosing at 10 mg/kg. These receptors are pharmacologically distinct, and previous studies have demonstrated that 10 mg/kg is sufficient to elicit measurable effects without the need for escalation. The dose of 30 mg/kg for the H4 receptor antagonist JNJ7777120 was chosen based on experimental evidence indicating that this dose provides more consistent pharmacological efficacy in rats. Coruzzi et al. [124] evaluated JNJ7777120 at both 10 mg/kg and 30 mg/kg in a carrageenan-induced inflammation model. While some effects were observed at the lower dose, the higher dose produced stronger and more consistent inhibition of paw edema and nociceptive responses. Therefore, 30 mg/kg was selected to ensure effective H4 receptor antagonism and to capture the potential anti-inflammatory and antioxidative actions relevant to our colitis model.

In summary, the chosen dosing strategies for TNBS, the histamine receptor antagonists, and the anesthetic protocol are based on established literature and pharmacological considerations, ensuring efficacy and reliability in the rat model.

### 4.4. Tissue Sampling and Preparation for Biochemical Analyses

Liver tissue samples were collected from the middle part of each liver lobe. Muscle tissue samples were collected from the soleus muscle (*musculus soleus*). The sampled tissue was weighed and homogenized in 0.9% NaCl on an ice bath using an Omni TH homogenizer with Omni Hard Tissue Tips (Omni International, Kennesaw, GA, USA). Then, the samples were centrifuged at 10,000× *g* for 10 min at 4 °C. The obtained homogenates were 10%. The collected supernatant was divided into subsamples and stored at −80 °C until needed for analyses.

### 4.5. Biochemical Analyses

All biochemical analyses were performed using a PERKIN ELMER Victor *X*3 reader (PerkinElmer, Inc., Waltham, MA, USA) or BioTek Synergy H1 Multimode Reader (Agilent, Santa Clara, CA, USA).

#### 4.5.1. Superoxide Dismutase (SOD) (EC 1.15.1.1) Activity

Superoxide dismutase activity was determined using the Oyanagui method [129] and was expressed as nitrite units (NU) per 1 min per 1 mg of protein in the tested sample, where 1 NU equals 50% inhibition of nitrite ion formation under the method’s conditions.

#### 4.5.2. Catalase (CAT) Activity

Catalase activity was assessed using the Aebi method [130] and was expressed as international units per 1 mg of protein (IU/mg protein) of the tested sample.

#### 4.5.3. Glutathione S-Transferase (GST) Activity

Glutathione S-transferase activity was determined using the kinetic method of Habig and Jakoby [131] and expressed in nmol of 1,2-dichloro-4-nitrobenzene (CDNB) over 1 min per 1 mg of protein of the tested sample.

#### 4.5.4. Reduced Glutathione (GSH) Concentration

Reduced glutathione concentration was determined using the Kobayashi et al. method [132], which measures the fluorescence at (λ_ex_: 355 nm and λ_em_: 420 nm), and expressed as μg of GSH per mg protein of the tested sample.

#### 4.5.5. Malondialdehyde (MDA) Concentration

Malondialdehyde concentration was measured using the method described by Ohkawa et al. [133], calculated against a standard curve prepared with thiobarbituric acid (TBA), and expressed as nmol TBA per mg of protein in the tested sample.

#### 4.5.6. Total Antioxidant Capacity (TAC)

Total antioxidant capacity was measured using Erel’s method [134] with 2,2′-azino-bis(3-ethylbenzothiazoline-6-sulfonate) (ABTS+), having blue-green color when oxidized or having no color when being reduced, and was expressed as µM of Trolox per mg protein of the tested sample.

#### 4.5.7. Total Oxidative Status (TOS)

Total oxidative status was determined using Erel’s method [135], and was expressed as µM of H_2_O_2_ per mg protein of the tested sample.

#### 4.5.8. α- and β-Esterase (EST) Activity

Esterase activity was determined using the kinetic method as described by van Asperen [136], using α- and β-naphthol and their respective absorbances, 600 nm for α-naphthol and 555 nm for β-naphthol, as substrates for respective specific activities. Their activity was calculated from the standard curve and expressed as nM of naphthol per minute per milligram of protein in the tested sample.

#### 4.5.9. Protein Concentration

Protein concentration was measured using the Bradford method [137].

### 4.6. Statistical Analysis

All statistical analyses were performed using Statistica 13 software (StatSoft Polska, Kraków, Poland). The results are presented as medians along with the lower and upper quartiles (Me [Q1–Q3]). The Kruskal–Wallis ANOVA was used to compare differences within the healthy and diseased groups based on the applied antagonists. To assess the effect of each specific receptor antagonist on the analyzed variable, the Mann–Whitney U test was applied. All tests were two-tailed, and statistical significance was set at *p* < 0.05.

## 5. Conclusions

This study demonstrates that blocking histamine receptors exerts receptor-specific and tissue-dependent effects on oxidative stress regulation in rats with TNBS-induced colitis. Blocking H1 and H2 receptors (with cetirizine and ranitidine, respectively) enhanced antioxidant defenses—evidenced by increased SOD, CAT, and GSH activities in colitis controls—and partially preserved redox balance under inflammatory conditions. Overall, the results indicate that histamine signaling modulates systemic oxidative homeostasis across both liver and skeletal muscle tissues during intestinal inflammation. The results highlight the H2 receptor as the most promising pharmacological target for attenuating colitis-associated oxidative injury, while blocking the H1 receptor may provide complementary benefits in preserving antioxidant enzyme activity. Collectively, histamine receptor modulation emerges as a potential therapeutic strategy to mitigate systemic oxidative stress and prevent extraintestinal complications in inflammatory bowel disease.

## Figures and Tables

**Table 1:** Oxidative stress markers in the liver tissue of adult male Wistar rats (*Rattus norvegicus*) non-treated (NT), treated with histamine H1 (cetirizine), H2 (ranitidine), H3 (iodophenpropit), or H4 (JNJ7777120) receptor antagonists from the colitis control (ColCtrl) groups and groups with chemically induced colitis ulcerosa.

Oxidative Stress Marker	Group	ColCtrl	Colitis	P_ColCtrl vs. Colitis_
SOD (NU/min/mg protein)	NT	2.8 (2.2–2.9)	4.2 (3.9–4.7)	<0.01
	H1	3.8 (3.2–4.0)	2.2 (2.2–2.4)	<0.01
	H2	4.9 (4.9–5.1)	3.0 (2.6–3.1)	<0.01
	H3	2.05 (2.0–2.1)	2.5 (2.3–2.6)	<0.05
	H4	2.5 (2.4–2.6)	2.4 (2.3–2.5)	0.575
CAT (U/mg protein/min)	NT	2.1 (1.8–2.7)	6.7 (6.5–6.8	<0.01
	H1	8.6 (8.2–10.0)	4.8 (4.7–5.0)	<0.01
	H2	14.4 (14.4–14.8)	6.2 (6.1–6.2)	<0.01
	H3	4.5 (4.2–4.8)	2.5 (2.3–2.7)	<0.01
	H4	3.4 (2.3–3.8)	1.7 (1.2–2.0)	<0.01
GST (nmol/mg protein/min)	NT	9.5 (9.4–9.9)	33.4 (33.2–33.6)	<0.01
	H1	33.2 (33.1–33.4)	16.6 (16.3–16.9)	<0.01
	H2	23.4 (23.1–23.7)	12.2 (12.1–12.3)	<0.01
	H3	8.9 (8.8–9.0)	17.0 (16.9–17.0)	<0.01
	H4	10.6 (10.5–10.7)	14.4 (14.1–14.7)	<0.01
GSH (µg/mg protein)	NT	0.69 (0.68–0.70)	1.11 (1.04–1.19)	<0.01
	H1	2.57 (2.42–2.63)	0.80 (0.79–0.84)	<0.01
	H2	0.81 (0.79–0.93)	0.47 (0.46–0.47)	<0.01
	H3	0.76 (0.73–0.80)	0.66 (0.66–0.66)	0.055
	H4	0.62 (0.62–0.65)	0.87 (0.82–0.94)	<0.01
MDA (nmol/mg protein)	NT	1.5 (1.3–1.8)	1.6 (1.4–2.1)	0.575
	H1	1.8 (1.7–1.8)	1.6 (1.5–1.8)	0.378
	H2	1.3 (1.2–1.5)	2.1 (2.0–2.6)	<0.01
	H3	1.4 (1.3–1.5)	2.0 (1.9–2.1)	<0.01
	H4	2.0 (1.9–2.4)	2.5 (2.3–2.7)	0.128
TAC (μM Trolox/mg protein)	NT	0.178 (0.177–0.184)	0.127 (0.120–0.128)	<0.01
	H1	0.182 (0.170–0.184)	0.164 (0.162–0.166)	0.298
	H2	0.191 (0.180–0.196)	0.264 (0.255–0.268)	<0.01
	H3	0.243 (0.203–0.283)	0.285 (0.281–0.287)	0.121
	H4	0.206 (0.198–0.209)	0.237 (0.227–0.260)	<0.05
TOS (µM H_2_O_2_/mg protein)	NT	0.044 (0.040–0.046)	0.029 (0.027–0.033)	<0.01
	H1	0.044 (0.044–0.046)	0.040 (0.033–0.043)	<0.01
	H2	0.032 (0.032–0.039)	0.041 (0.039–0.045)	0.066
	H3	0.042 (0.039–0.046)	0.047 (0.043–0.048)	0.121
	H4	0.049 (0.047–0.055)	0.046 (0.042–0.047)	0.173
α-EST (nM/min/mg protein)	NT	2.62 (2.32–2.70)	1.29 (0.98–1.46)	<0.01
	H1	2.29 (2.27–2.34)	1.25 (1.18–1.66)	<0.01
	H2	2.18 (1.98–2.96)	1.91 (1.26–2.08)	0.128
	H3	2.04 (1.88–2.16)	1.70 (1.67–1.76)	0.055
	H4	2.33 (2.21–2.36)	2.08 (1.78–2.37)	0.378
β-EST (nM/min/mg protein)	NT	0.124 (0.120–0.129)	0.204 (0.163–0.259)	<0.01
	H1	0.238 (0.231–0.248)	0.063 (0.060–0.064)	<0.01
	H2	0.229 (0.209–0.264)	0.063 (0.060–0.085)	<0.01
	H3	0.114 (0.112–0.115)	0.056 (0.056–0.058)	<0.01
	H4	0.056 (0.051–0.059)	0.067 (0.063–0.070)	<0.05

P—U Mann–Whitney test, P_ColCtrl vs. Colitis_—comparison between colitis control (ColCtrl) and colitis rats within each treatment group. Legend: CAT—catalase activity, EST—esterase activity, GSH—glutathione concentration, GST—glutathione S-transferase, MDA—malondialdehyde concentration, SOD—superoxide dismutase activity, TAC—total antioxidant activity, TOS—total oxidative status.

**Table 2:** Comparison of oxidative stress markers in the liver tissue of adult male Wistar rats (*Rattus norvegicus*) non-treated (NT), treated with histamine H1 (cetirizine), H2 (ranitidine), H3 (iodophenpropit), or H4 (JNJ7777120) receptor antagonists from the colitis control (ColCltr) groups and groups with chemically induced colitis ulcerosa.

Oxidative Stress Marker	Group	P_Kruskal-Wallis_	P_NT vs. H1_	P_NT vs. H2_	P_NT vs. H3_	P_NT vs. H4_
SOD	ColCtrl	<0.001	0.766	<0.05	1.0	1.0
	Colitis	<0.001	<0.01	1.0	<0.05	<0.01
CAT	ColCtrl	<0.001	<0.01	<0.001	0.182	1.0
	Colitis	<0.001	0.191	1.0	<0.01	<0.001
GST	ColCtrl	<0.001	<0.01	0.182	1.0	1.0
	Colitis	<0.001	0.386	<0.001	1.0	<0.01
GSH	ColCtrl	<0.001	<0.01	0.182	1.0	1.0
	Colitis	<0.001	0.386	<0.001	1.0	<0.01
MDA	ColCtrl	<0.01	1.0	1.0	1.0	0.105
	Colitis	<0.01	1.0	0.492	1.0	<0.05
TAC	ColCtrl	<0.001	1.0	1.0	<0.01	0.087
	Colitis	<0.001	1.0	<0.01	<0.001	<0.05
TOS	ColCtrl	<0.01	1.0	0.359	1.0	0.713
	Colitis	<0.05	1.0	1.0	0.087	0.090
β-EST	ColCtrl	0.174	-	-	-	-
	Colitis	<0.01	1.0	0.300	0.351	<0.05
α-EST	ColCtrl	<0.001	0.663	0.881	1.0	0.182
	Colitis	<0.001	<0.05	0.090	<0.001	0.356

P_NT vs. H1–H4_—comparison between non-treated (NT) and histamine receptor antagonist-treated rats within the colitis control (ColCtrl) or colitis group. Legend: CAT—catalase activity, EST—esterase activity, GSH—glutathione concentration, GST—glutathione S-transferase, MDA—malondialdehyde concentration, SOD—superoxide dismutase activity, TAC—total antioxidant activity, TOS—total oxidative status.

**Table 3:** Oxidative stress markers in the soleus muscle of adult male Wistar rats (*Rattus norvegicus*) non-treated (NT), treated with histamine H1 (cetirizine), H2 (ranitidine), H3 (iodophenpropit), or H4 (JNJ7777120) receptor antagonists from the colitis control (ColCtrl) groups and groups with chemically induced colitis ulcerosa.

Oxidative Stress Marker	Group	ColCtrl	Colitis	P_ColCtrl vs. Colitis_
SOD (NU/min/mg protein)	NT	6.121 (5.056–6.345)	14.547 (11.516–18.998)	<0.05
	H1	15.541 (15.088–16.671)	5.632 (4.979–6.848)	<0.01
	H2	15.417 (15.296–15.777)	6.331 (4.699–7.333)	<0.01
	H3	5.136 (4.419–5.328)	6.171 (5.960–9.192)	0.235
	H4	8.461 (6.839–8.910)	5.828 (5.147–6.813)	0.173
CAT (U/mg protein/min)	NT	1.516 (1.376–1.587)	8.426 (6.509–8.8816)	<0.01
	H1	6.355 (6.276–6.481)	1.351 (0.588–2.104)	<0.01
	H2	6.185 (5.948–6.474)	5.677 (5.602–13.298)	0.927
	H3	2.899 (2.288–3.571)	2.611 (2.105–4.697)	1.0
	H4	1.887 (1.048–2.496)	3.195 (2.876–3.469)	<0.05
GST (nmol/mg protein/min)	NT	3.424 (3.228–3.623)	3.892 (3.278–4.305)	0.173
	H1	6.334 (6.243–3.368)	3.804 (3.328–4.125)	<0.01
	H2	4.618 (4.397–5.131)	2.959 (2.503–3.601)	<0.01
	H3	3.294 (3.233–3.480)	4.179 (3.143–4.907)	0.523
	H4	4.342 (3.936–4.873)	4.390 (2.703–4.809)	0.689
GSH (µg/mg protein)	NT	0.354 (0.349–0.397)	0.964 (0.710–1.512)	<0.01
	H1	1.461 (1.134–1.695)	0.403 (0.336–0.454)	<0.01
	H2	1.697 (1.188–1.812)	0.685 (0.501–0.753)	<0.01
	H3	0.365 (0.338–0.381)	0.541 (0.299–0.847)	0.648
	H4	0.602 (0.524–0.634)	0.507 (0.171–0.582)	0.128
MDA (nmol/mg protein)	NT	0.302 (0.220–0.399)	0.234 (0.149–0.614)	0.575
	H1	0.316 (0.312–0.328)	0.141 (0.097–0.164)	<0.01
	H2	0.250 (0.222–0.261)	0.124 (0.107–0.157)	0.066
	H3	0.163 (0.131–0.182)	0.197 (0.194–0.330)	0.235
	H4	0.394 (0.241–0.887)	0.249 (0.218–0.330)	0.173
TAC (μM Trolox/mg protein)	NT	0.073 (0.072–0.078)	0.270 (0.225–0.343)	<0.01
	H1	0.256 (0.222–0.276)	0.043 (0.025–0.069)	<0.05
	H2	0.231 (0.222–0.281)	0.074 (0.054–0.078)	<0.01
	H3	0.046 (0.016–0.058)	0.026 (0.025–0.054)	<0.001
	H4	0.042 (0.041–0.044)	0.037 (0.033–0.040)	0.230
TOS (µM H_2_O_2_/mg protein)	NT	0.093 (0.093–0.094)	0.016 (0.015–0.041)	<0.01
	H1	0.012 (0.011–0.019)	0.002 (0.002–0.003)	<0.01
	H2	0.056 (0.015–0.094)	0.003 (0.003–0.005)	<0.01
	H3	0.028 (0.013–0.042)	0.057 (0.056–0.071)	<0.01
	H4	0.074 (0.073–0.075)	0.084 (0.084–0.085)	<0.01
α-EST (nM/min/mg protein)	NT	0.168 (0.151–0.181)	0.074 (0.049–0.092)	<0.05
	H1	0.149 (0.135–0.171)	0.063 (0.053–0.079)	<0.01
	H2	0.234 (0.182–0.294)	0.156 (0.102–0.196)	0.066
	H3	0.149 (0.111–0.191)	0.177 (0.175–0.183)	0.927
	H4	0.180 (0.161–0.189)	0.169 (0.117–0.179)	0.378
β-EST (nM/min/mg protein)	NT	0.091 (0.090–0.093)	0.017 (0.016–0.020)	<0.01
	H1	0.018 (0.016–0.022)	0.080 (0.075–0.083)	<0.01
	H2	0.015 (0.015–0.022)	0.072 (0.71–0.082)	<0.01
	H3	0.082 (0.015–0.093)	0.072 (0.072–0.074)	0.523
	H4	0.011 (0.010–0.011)	0.093 (0.081–0.096)	<0.01

P—U Mann–Whitney test, P_ColCtrl vs. Colitis_—comparison between colitis control (ColCtrl) and colitis rats within each treatment group. Legend: CAT—catalase activity, EST—esterase activity, GSH—glutathione concentration, GST—glutathione S-transferase, MDA—malondialdehyde concentration, SOD—superoxide dismutase activity, TAC—total antioxidant activity, TOS—total oxidative status.

**Table 4:** Comparison of oxidative stress markers in the soleus muscle of adult male Wistar rats (*Rattus norvegicus*) non-treated (NT), treated with histamine H1 (cetirizine), H2 (ranitidine), H3 (iodophenpropit), or H4 (JNJ7777120) receptor antagonists from the colitis control (ColCltr) groups and groups with chemically induced colitis ulcerosa.

Oxidative Stress Marker	Group	P_Kruskal-Wallis_	P_NT vs. H1_	P_NT vs. H2_	P_NT vs. H3_	P_NT vs. H4_
SOD	ColCtrl	<0.001	<0.05	<0.05	1.0	1.0
	Colitis	<0.05	0.077	0.138	0.225	0.291
CAT	ColCtrl	<0.001	<0.01	<0.01	1.0	1.0
	Colitis	<0.001	<0.01	1.0	0.103	0.464
GST	ColCtrl	<0.001	<0.001	0.182	1.0	0.530
	Colitis	0.268	-	-	-	-
GSH	ColCtrl	<0.001	<0.01	<0.01	1.0	0.713
	Colitis	0.054	-	-	-	-
MDA	ColCtrl	<0.01	1.0	1.0	0.059	1.0
	Colitis	<0.05	0.386	0.724	1.0	1.0
TAC	ColCtrl	<0.001	1.0	1.0	0.945	0.766
	Colitis	<0.01	<0.05	0.455	<0.01	<0.01
TOS	ColCtrl	<0.001	<0.01	1.0	<0.05	1.0
	Colitis	<0.001	0.082	0.900	1.0	0.848
β-EST	ColCtrl	<0.01	0.270	0.286	1.0	<0.01
	Colitis	<0.001	<0.05	0.175	1.0	<0.001
α-EST	ColCtrl	<0.05	1.0	0.388	1.0	1.0
	Colitis	<0.01	1.0	0.121	0.092	0.160

P_NT vs. H1–H4_—comparison between non-treated (NT) and histamine receptor antagonist treated rats within the colitis control (ColCtrl) or colitis group. Legend: CAT—catalase activity, EST—esterase activity, GSH—glutathione concentration, GST—glutathione S-transferase, MDA—malondialdehyde concentration, SOD—superoxide dismutase activity, TAC—total antioxidant activity, TOS—total oxidative status.

## Data Availability

The original contributions presented in this study are included in the article. Further inquiries can be directed to the corresponding author.

## References

[1] Caron B., Honap S., Peyrin-Biroulet L. (2024). Epidemiology of inflammatory bowel disease across the ages in the era of advanced therapies. J. Crohn’s Colitis.

[2] Ng S.C., Shi H.Y., Hamidi N., Underwood F.E., Tang W., Benchimol E.I., Panaccione R., Ghosh S., Wu J.C.Y., Chan F.K.L. (2017). Worldwide incidence and prevalence of inflammatory bowel disease in the 21st century: A systematic review of population-based studies. Lancet.

[3] Torres J., Mehandru S., Colombel J.F., Peyrin-Biroulet L. (2017). Crohn’s disease. Lancet.

[4] Kaser A., Zeissig S., Blumberg R.S. (2010). Inflammatory bowel disease. Ann. Rev. Immunol..

[5] Neurath M.F. (2020). Current and emerging therapeutic targets for IBD. Nat. Rev. Gastroenterol. Hepatol..

[6] Roda G., Ng S.C., Kotze P.G., Argollo M., Panaccione R., Spinelli A., Kaser A., Peyrin-Biroulet L., Danese S. (2020). Crohn’s disease. Nat. Rev. Dis. Primers.

[7] Papa A., Scaldaferri F., Danese S., Guglielmo S., Roberto I., Bonizzi M., Mocci G., Felice C., Ricci C., Andrisani G. (2008). Vascular involvement in inflammatory bowel disease: Pathogenesis and clinical aspects. Dig. Dis..

[8] Kotlyar D.S., Shum M., Hsieh J., Blonski W., Greenwald D.A. (2014). Non-pulmonary allergic diseases and inflammatory bowel disease: A qualitative review. World J. Gastroenterol..

[9] Smolinska S., Winiarska E., Globinska A., Jutel M. (2022). Histamine: A mediator of intestinal disorders-a review. Metabolites.

[10] Ashina K., Tsubosaka Y., Nakamura T., Omori K., Hori M., Ozaki H., Murata T. (2015). Histamine induces vascular hyperpermeability by increasing blood flow and endothelial barrier disruption in vivo. PLoS ONE.

[11] Wunschel E.J., Schirmer B., Seifert R., Neumann D. (2017). Lack of histamine H4-receptor expression aggravates TNBS-induced acute colitis symptoms in mice. Front. Pharmacol..

[12] Fogel W.A., Wagner W., Stasiak K. (2005). The role of histamine in experimental ulcerative colitis in rats. Inflamm. Res..

[13] Daugherty B.L. (2004). Histamine H4 antagonism: A therapy for chronic allergy?. Br. J. Pharmacol..

[14] Gao C., Major A., Rendon D., Lugo M., Jackson V., Shi Z., Mori-Akiyama Y., Versalovic J. (2015). Histamine H2 receptor-mediated suppression of intestinal inflammation by probiotic *Lactobacillus reuteri*. mBio.

[15] Deiteren A., De Man J.G., Ruyssers N.E., Moreels T.G., Pelckmans P.A., De Winter B.Y. (2014). Histamine H4 and H1 receptors contribute to postinflammatory visceral hypersensitivity. Gut.

[16] Deiteren A., De Man J.G., Pelckmans P.A., De Winter B.Y. (2015). Histamine H_4_ receptors in the gastrointestinal tract. Br. J. Pharmacol..

[17] Okayama M., Tsubouchi R., Kato S., Takeuchi K. (2004). Protective effect of lafutidine, a novel histamine H2-receptor antagonist, on dextran sulfate sodium-induced colonic inflammation through capsaicin-sensitive afferent neurons in rats. Dig. Dis. Sci..

[18] Fogel W.A., Jochem J., Lewinski A. (2007). Influence of the H3/H4 receptor antagonist, thioperamide on regional haemodynamics in rats with trinitrobenzene sulfonic acid-induced colitis. Inflamm. Res..

[19] Wechsler J.B., Szabo A., Hsu C.L., Kreir-Burris R.A., Schroede H.A., Wang M.Y., Carter R.G., Velez T.E., Aguiniga L.M., Brown J.B. (2018). Histamine drives severity of innate inflammation via histamine 4 receptor in murine experimental colitis. Mucosal Immunol..

[20] Guth P.H., Smith E. (1978). Histamine receptors in mesenteric circulation of the cat and rat. Am. J. Physiol.-Endocrinol. Metab..

[21] Shahinian H.K., Ferguson M.K., Michelassi F. (1987). Effect of histamine and histamine receptor antagonists on rat mesenteric microcirculation. J. Surg. Res..

[22] Obuchowicz M., Pawlik M.W., Brzozowski T., Konturek S.J., Pawli W.W. (2004). Involvement of central and peripheral histamine H3 receptors in the control of the vascular tone and oxygen uptake in the mesenteric circulation of the rat. J. Physiol. Pharmacol..

[23] Charbon G.A., Brouwers H.A., Sala A. (1980). Histamine H1- and H2-receptors in the gastrointestinal circulation. Naunyn-Schmiedeberg’s Arch. Pharmacol..

[24] Jin H., Koyama T., Hatanaka Y., Akiyama S., Takayama F., Kawasaki H. (2006). Histamine-induced vasodilation and vasoconstriction in the mesenteric resistance artery of the rat. Eur. J. Pharmacol..

[25] Pawlik W.W., Obuchowicz R., Pawlik M.W., Sendur R., Biernat J., Brzozowski T., Konturek S.J. (2004). Histamine H3 receptors modulate reactive hyperemia in rat gut. J. Physiol. Pharmacol..

[26] Pilz P.M., Ward J.E., Chang W.T., Kiss A., Bateh E., Jha A., Fisch S., Podesser B.K., Liao R. (2022). Large and small animal models of heart failure with reduced ejection fraction. Circ. Res..

[27] Leong X.F., Ng C.Y., Jaarin K. (2015). Animal models in cardiovascular research: Hypertension and atherosclerosis. BioMed Res. Int..

[28] Homberg J.R., Wöhr M., Alenina N. (2017). Comeback of the rat in biomedical research. ACS Chem. Neurosci..

[29] Pfeffer M.A., Pfeffer J.M., Fishbein M.C., Fletcher P.J., Spadaro J., Kloner R.A., Braunwald E. (1979). Myocardial infarct size and ventricular function in rats. Circ. Res..

[30] Patten R.D., Hall-Porter M.R. (2009). Small animal models of heart failure: Development of novel therapies, past and present. Circ. Heart Fail..

[31] Zhao Y., Qu H., Wang Y., Xiao W., Zhang Y., Shi D. (2020). Small rodent models of atherosclerosis. Biomed. Pharmacother..

[32] Veskoukis A.S., Goutianos G., Paschalis V., Margaritelis N.V., Tzioura A., Dilpa K., Zafeiridis A., Vrabas I.S., Kyparos A., Nikolaidis M.G. (2016). The rat closely mimics oxidative stress and inflammation in humans after exercise but not after exercise combined with vitamin C administration. Eur. J. Appl. Physiol..

[33] Tran V., De Silva T.M., Sobey C.G., Lim K., Drummond G.R., Vinh A., Jelinic M. (2020). The vascular consequences of metabolic syndrome: Rodent models, endothelial dysfunction, and current therapies. Front. Pharmacol..

[34] Antoniou E., Margonis G.A., Angelou A., Pikouli A., Argiri P., Karavokyros I., Papalois A., Pikoulis E. (2016). The TNBS-induced colitis animal model: An overview. Ann. Med. Surg..

[35] Silva I., Solas J., Pinto R., Mateus V. (2022). Chronic experimental model of TNBS-induced colitis to study inflammatory bowel disease. Int. J. Mol. Sci..

[36] Wirtz S., Neufert C., Weigmann B., Neurath M.F. (2007). Chemically induced mouse models of intestinal inflammation. Nat. Protoc..

[37] Morris G.P., Beck P.L., Herridge M.S., Depew W.T., Szewczuk M.R., Wallace J.L. (1989). Hapten-induced model of chronic inflammation and ulceration in the rat colon. Gastroenterology.

[38] Kiesler P., Fuss I.J., Strober W. (2015). Experimental models of inflammatory bowel diseases. Cell. Mol. Gastroenterol. Hepatol..

[39] Wen C., Chen D., Zhong R., Peng X. (2024). Animal models of inflammatory bowel disease: Category and evaluation indexes. Gastroenterol. Rep..

[40] Wirtz S., Popp V., Kindermann M., Gerlach K., Weigmann B., Fichtner-Feigl S., Neurath M.F. (2017). Chemically induced mouse models of acute and chronic intestinal inflammation. Nat. Protoc..

[41] Laroui H., Ingersoll S.A., Liu H.C., Baker M.T., Ayyadurai S., Charania M.A., Laroui F., Yan Y., Sitaraman S.V., Merlin D. (2012). Dextran sodium sulfate (DSS) induces colitis in mice by forming nano-lipocomplexes with medium-chain-length fatty acids in the colon. PLoS ONE.

[42] Sui Y., Jiang R., Niimi M., Wang X., Xu Y., Zhang Y., Shi Z., Suda M., Mao Z., Fan J. (2024). Gut bacteria exacerbates TNBS-induced colitis and kidney injury through oxidative stress. Redox Biol..

[43] Ranganathan P., Jayakumar C., Manicassamy S., Ramesh G. (2013). CXCR2 knockout mice are protected against DSS-colitis-induced acute kidney injury and inflammation. Am. J. Physiol.-Ren. Physiol..

[44] Knauf F., Brewer J.R., Flavell R.A. (2019). Immunity, microbiota and kidney disease. Nat. Rev. Nephrol..

[45] Wang Y., Duan X., Liu X., Liu X., Fan H., Xu M., Chen Q., Tang Q. (2020). Rho kinase blockade ameliorates DSS-induced ulcerative colitis in mice through dual inhibition of the NF-κB and IL-6/STAT3 pathways. Inflammation.

[46] Hambardikar V.R., Mandlik D.S. (2022). Protective effect of naringin ameliorates TNBS-induced colitis in rats via improving antioxidant status and pro-inflammatory cytokines. Immunopharmacol. Immunotoxicol..

[47] Almási N., Török S., Al-Awar A., Veszelka M., Kiraly L., Borzsei D., Szabo R., Varga C. (2023). Voluntary exercise-mediated protection in TNBS-induced rat colitis: The involvement of NETosis and Prdx antioxidants. Antioxidants.

[48] Jena G., Trivedi P.P., Sandala B. (2012). Oxidative stress in ulcerative colitis: An old concept but a new concern. Free Radic. Res..

[49] Wang W., Han Y., Yin W., Wang Q., Wu Y., Du M. (2024). Intestinal and hepatic benefits of BBR-EVO on DSS-induced experimental colitis in mice. Front. Microbiol..

[50] Trivedi P.P., Jena G.B. (2013). Ulcerative colitis-induced hepatic damage in mice: Studies on inflammation, fibrosis, oxidative DNA damage and GST-P expression. Chem. Biol. Interact..

[51] da Paz Martins A.S., de Andrade K.Q., de Araújo O.R.P., da Conceição G.C.M., da Silva Gomes A., Goulart M.O.F., Moura F.A. (2023). Extraintestinal manifestations in induced colitis: Controversial effects of *N*-acetylcysteine on colon, liver, and kidney. Oxidative Med. Cell. Longev..

[52] Hu L.H., Liu J.Y., Yin J.B. (2021). Eriodictyol attenuates TNBS-induced ulcerative colitis through repressing TLR4/NF-kB signaling pathway in rats. Kaohsiung J. Med. Sci..

[53] da Paz Martins A.S., Gomes Campos S.B., Fonseca Goulart M.O., Moura F.A. (2021). Extraintestinal manifestations of inflammatory bowel disease, nitroxidative stress and dysbiosis: What is the link between them?. Biocell.

[54] Brooks A.C., Whelan C.J., Purcell W.M. (1999). Reactive oxygen species generation and histamine release by activated mast cells: Modulation by nitric oxide synthase inhibition. Br. J. Pharmacol..

[55] Akamatsu H., Miyachi Y., Asada Y., Niwa Y. (1991). Effects of azelastine on neutrophil chemotaxis, phagocytosis and oxygen radical generation. Jpn. J. Pharmacol..

[56] Zhou E., Wu Z., Zhu X., Li P., Wang J., Yang Z. (2021). Histamine triggers the formation of neutrophil extracellular traps via NADPH oxidase, ERK and p38 pathways. Vet. Immunol. Immunopathol..

[57] Simons F.E., Simons K.J. (1994). The pharmacology and use of H1-receptor-antagonist drugs. N. Engl. J. Med..

[58] Church D.S., Church M.K. (2011). Pharmacology of antihistamines. World Allergy Organ. J..

[59] Parsons M.E., Ganellin C.R. (2006). Histamine and its receptors. Br. J. Pharmacol..

[60] Leurs R., Blandina P., Tedford C., Timmerman H. (1998). Therapeutic potential of histamine H3 receptor agonists and antagonists. Trends Pharmacol. Sci..

[61] Lim H.D., van Rijn R.M., Ling P., Bakker R.A., Thurmond R.L., Leurs R. (2005). Evaluation of histamine H1-, H2-, and H3-receptor ligands at the human histamine H4 receptor: Identification of 4-methylhistamine as the first potent and selective H4 receptor agonist. J. Pharmacol. Exp. Ther..

[62] Thurmond R.L., Desai P.J., Dunford P.J., Fung-Leung W.P., Hofstra C.L., Jiang W., Nguyen S., Riley J.P., Sun S., Williams K.N. (2004). A potent and selective histamine H4 receptor antagonist with anti-inflammatory properties. J. Pharmacol. Exp. Ther..

[63] Jójárt B., Kiss R., Viskolcz B., Keseru G.M. (2008). Activation mechanism of the human histamine H4 receptor--an explicit membrane molecular dynamics simulation study. J. Chem. Inf. Model..

[64] Francis H., Meng F., Gaudio E., Alpini G. (2012). Histamine regulation of biliary proliferation. J. Hepatol..

[65] Tripathi T., Shahid M., Khan H.M., Khan R.A., Siddiqui M., Mahdi A.A. (2011). The Influence of histamine H1-receptor on liver functions in immunized rabbits. Saudi J. Biol. Sci..

[66] Patel R.H., Mohiuddin S.S. (2023). Biochemistry, Histamine. StatPearls.

[67] Francis H., Franchitto A., Ueno Y., Glaser S., DeMorrow S., Venter J., Gaudio E., Alvaro D., Fava G., Marzioni M. (2007). H3 histamine receptor agonist inhibits biliary growth of BDL rats by downregulation of the cAMP-dependent PKA/ERK1/2/ELK-1 pathway. Lab. Investig..

[68] Héron A., Rouleau A., Cochois V., Pillot C., Schwartz J.C., Arrang J.M. (2001). Expression analysis of the histamine H(3) receptor in developing rat tissues. Mech. Dev..

[69] Speeg K.V., Patwardhan R.V., Avant G.R., Mitchell M.C., Schenker S. (1982). Inhibition of microsomal drug metabolism by histamine H2-receptor antagonists studied in vivo and in vitro in rodents. Gastroenterology.

[70] Adachi N., Liu K., Motoki A., Nishibori M., Arai T. (2006). Suppression of ischemia/reperfusion liver injury by histamine H4 receptor stimulation in rats. Eur. J. Pharmacol..

[71] Cowden J.M., Yu F., Challapalli M., Huang J.F., Kim S., Fung-Leung W.P., Ma J.Y., Riley J.P., Zhang M., Dunford P.J. (2013). Antagonism of the histamine H4 receptor reduces LPS-induced TNF production in vivo. Inflamm. Res..

[72] Schirmer B., Neumann D., Seifert R. (2021). Analysis of histamine receptor knockout mice in models of inflammation. J. Pharmacol. Exp. Ther..

[73] (2018). LiverTox: Clinical and Research Information on Drug-Induced Liver Injury [Internet]. Bethesda (MD): National Institute of Diabetes and Digestive and Kidney Diseases; 2012-. Histamine Type-2 Receptor Antagonists (H2 Blockers). https://www.ncbi.nlm.nih.gov/books/NBK547929.

[74] Kasimay O., Güzel E., Gemici A., Abdyli A., Sulovari A., Ercan F., Yegen B.C. (2006). Colitis-induced oxidative damage of the colon and skeletal muscle is ameliorated by regular exercise in rats: The anxiolytic role of exercise. Exp. Physiol..

[75] Novak E.A., Mollen K.P. (2015). Mitochondrial dysfunction in inflammatory bowel disease. Front. Cell Dev. Biol..

[76] Metzger C.E., Narayanan S.A., Elizondo J.P., Elizondo J.P., Carter A.M., Zawieja D.C., Hogan H.A., Bloomfield S.A. (2019). DSS-induced colitis produces inflammation-induced bone loss while irisin treatment mitigates the inflammatory state in both gut and bone. Sci. Rep..

[77] Kim J.H., Lawler J.M. (2012). Amplification of proinflammatory phenotype, damage, and weakness by oxidative stress in the diaphragm muscle of mdx mice. Free Radic. Biol. Med..

[78] Moylan J.S., Reid M.B. (2007). Oxidative stress, chronic disease, and muscle wasting. Muscle Nerve.

[79] González García A., Sifuentes-Giraldo W.A., Diz Fariña S., Pian H. (2016). Polymyositis in a patient with ulcerative colitis. Reumatol. Clin..

[80] Nagi T.K., Gheit Y., Hernandez O.L., Suarez Z.K., Vallejo C., Haider M.A., Zahra T. (2023). Myositis as an extraintestinal manifestation of ulcerative colitis: A case report and literature review. Cureus.

[81] Saul D., Kosinsky R.L. (2020). Dextran sodium sulfate-induced colitis as a model for sarcopenia in mice. Inflamm. Bowel Dis..

[82] Nardone O.M., de Sire R., Petito V., Testa A., Villani G., Scaldaferri F., Castiglione F. (2021). Inflammatory bowel diseases and sarcopenia: The role of inflammation and gut microbiota in the development of muscle failure. Front. Immunol..

[83] Sieck D.C., Kobak S.H., Larson E.A., Dreyer H.C., Fogarty M.J., Sieck G.C., Minson C.T., Halliwill J.R. (2025). Histamine is a molecular transducer of adaptation to endurance exercise training in humans. J. Appl. Physiol..

[84] Dvornikova K.A., Platonova O.N., Bystrova E.Y. (2023). Inflammatory bowel disease: Crosstalk between histamine, immunity, and disease. Int. J. Mol. Sci..

[85] Wei Y., Zhang J., Yan X., Peng X., Xu S., Chang H., Wang H., Gao Y. (2019). Remarkable protective effects of Nrf2-mediated antioxidant enzymes and tissue specificity in different skeletal muscles of daurian ground squirrels over the torpor-arousal cycle. Front. Physiol..

[86] Tas S., Taş B., Bassalat N., Jaradat N.A. (2018). In-vivo, hypoglycemic, hypolipidemic, and oxidative stress inhibitory activities of Myrtus communis L. fruits hydroalcoholic extract in normoglycemic and streptozotocin-induced diabetic rats. Biomed Res.

[87] Bindels L.B., Porporato P., Dewulf E.M., Varrax J., Neyrinck A.M., Martin J.C., Scott K.P., Calderon P.B., Feron O., Mucciolo G.G. (2012). Gut microbiota-derived propionate reduces cancer cell proliferation in the liver. Br. J. Cancer.

[88] Huang L.J., Wang Y.M., Gong L.Q., Hu L.-Q., Gui Y., Zhang C., Tan X., Yu X.-K., Liao Y.-L., Luo Y. (2022). N-acetyldopamine dimer attenuates DSS-induced ulcerative colitis by suppressing NF-κB and MAPK pathways. Front. Pharmacol..

[89] Ji L.L. (2025). Nuclear factor κB signaling revisited: Its role in skeletal muscle and exercise. Free Radic. Biol. Med..

[90] González-Bosch C., Boorman E., Zunszain P.A., Mann G.E. (2021). Short-chain fatty acids as modulators of redox signaling in health and disease. Redox Biol..

[91] Liu P., Wang Y., Yang G., Zhang Q., Meng L., Xin Y., Jiang X. (2021). The role of short-chain fatty acids in intestinal barrier function, inflammation, oxidative stress, and colonic carcinogenesis. Pharmacol. Res..

[92] Hays K.E., Pfaffinger J.M., Ryznar R. (2024). The interplay between gut microbiota, short-chain fatty acids, and implications for host health and disease. Gut Microbes.

[93] Li T., Yin D., Shi R. (2024). Gut-muscle axis mechanism of exercise prevention of sarcopenia. Front. Nutr..

[94] Li W., Sheng R.W., Cao M.M., Rui Y.F. (2024). Exploring the relationship between gut microbiota and sarcopenia based on gut-muscle axis. Food Sci. Nutr..

[95] Powers S.K. (2014). Can antioxidants protect against disuse muscle atrophy?. Sports Med..

[96] El Assar M., Álvarez-Bustos A., Sosa P., Angulo J., Rodríguez-Mañas L. (2022). Effect of physical activity/exercise on oxidative stress and inflammation in muscle and vascular aging. Int. J. Mol. Sci..

[97] Huffman K.M., Jesse R., Andonian B., Davis B.N., Narowski R., Huebner J.L., Kraus V.B., McCracken J., Gilmore B.F., Tune K.N. (2017). Molecular alterations in skeletal muscle in rheumatoid arthritis are related to disease activity, physical inactivity, and disability. Arthritis Res. Ther..

[98] Chen M., Wang T., Deng J., Lian Z., Yu K. (2022). Skeletal muscle oxidative stress and inflammation in aging: Focus on antioxidant and anti-inflammatory therapy. Front. Cell Dev. Biol..

[99] Nieto-Alamilla G., Márquez-Gómez R., García-Gálvez A.M., Morlaes-Figueroa G.-E., Arias-Montano J.A. (2016). The histamine H3 receptor: Structure, pharmacology, and function. Mol. Pharmacol..

[100] Hanuskova E., Plevkova J. (2013). Histamine and its effects mediated via h3 receptor—potential clinical applications of h3 antagonists. Acta Med. Martin..

[101] Thurmond R.L., Gelfand E.W., Dunford P.J. (2008). The role of histamine H1 and H4 receptors in allergic inflammation: The search for new antihistamines. Nat. Rev. Drug Discov..

[102] Cowden J.M., Riley J.P., Ma J.Y., Thurmond R.L., Dunford P.J. (2010). Histamine H4 receptor antagonism diminishes existing airway inflammation and dysfunction via modulation of Th2 cytokines. Respir. Res..

[103] Abrigo J., Simon F., Cabrera D., Vilos C., Cabello-Verrugio C. (2019). Mitochondrial dysfunction in skeletal muscle pathologies. Curr. Protein Pept. Sci..

[104] Zhi J., Jiao B., Qing S., Liang L. (2023). Factors associated with low skeletal muscle index among patients with Crohn’s disease. Rev. Assoc. Médica Bras..

[105] Barnouin Y., McPhee J.S., Butler-Browne G., Bosutti A., De Vito G., Jones D.A., Narici M., Behin A., Hogrel J.-Y., Degens H. (2017). Coupling between skeletal muscle fiber size and capillarization is maintained during healthy aging. J. Cachexia Sarcopenia Muscle..

[106] Motil K.J., Grand R.J., Davis-Kraft L., Ferlic L.L., O-Brian Smith E. (1993). Growth failure in children with inflammatory bowel disease: A prospective study. Gastroenterology.

[107] Ueno M., Inagaki N., Nagai H., Koda A. (1998). Antiallergic action of betotastine besilate (TAU- 284) in animal models: A comparison with ketotifen. Pharmacology.

[108] Harada C., Fujii Y., Hirai T., Shinomiya K., Kamei C. (2004). Inhibitory effect of iodophenpropit, a selective histamine H3 antagonist, on amygdaloid kindled seizures. Brain Res. Bull..

[109] Fatani H., Olaru A., Stevenson R., Alharazi W., Jafer A., Atherton P., Brook M., Moran G. (2023). Systematic review of sarcopenia in inflammatory bowel disease. Clin. Nutr..

[110] Puleo F., Meirelles K., Navaratnarajah M., Fitzpatrick L., Shumate M.L., Cooney R.N., Lang C.H. (2010). Skeletal muscle catabolism in trinitrobenzene sulfonic acid-induced murine colitis. Metabolism.

[111] DeVoss J., Diehl L. (2014). Murine models of inflammatory bowel disease (IBD): Challenges of modeling human disease. Toxicol. Pathol..

[112] Sandborn W.J., Su C., Sands B.E., D’Haens G.R., Vermeire S., Schreiber S., Danese S., Feagan B.G., Reinisch W., Niezychowski W. (2017). Tofacitinib as induction and maintenance therapy for ulcerative colitis. N. Engl. J. Med..

[113] Danese S., Vermeire S., Zhou W., Pangan A.L., Siffledeen J., Greenbloom S., Hébuterne X., D’Haens G., Nakase H., Panés J. (2022). Upadacitinib as induction and maintenance therapy for moderately to severely active ulcerative colitis: Results from three phase 3, multicentre, double-blind, randomised trials. Lancet.

[114] O’Shea J.J., Schwartz D.M., Villarino A.V., Gadina M., McInnes I.B., Laurence A. (2015). The JAK-STAT pathway: Impact on human disease and therapeutic intervention. Annu. Rev. Med..

[115] Simon A.R., Rai U., Fanburg B.L., Cochran B.H. (1998). Activation of the JAK-STAT pathway by reactive oxygen species. American J. Physiol.-Cell Physiol..

[116] Manea A., Manea S.A., Florea I.C., Luca C.M., Raicu M. (2012). Positive regulation of NADPH oxidase 5 by proinflammatory-related mechanisms in human aortic smooth muscle cells. Free Radic. Biol. Med..

[117] Russell W.M.S., Burch R.L. (1959). The Principles of Humane Experimental Technique.

[118] Festing M.F.W., Altman D.G. (2002). Guidelines for the design and statistical analysis of experiments using laboratory animals. ILAR J..

[119] Percie du Sert N., Hurst V., Ahluwalia A., Alam S., Avey M.T., Baker M., Browne W.J., Clark A., Cuthill I.C., Dirnagl U. (2020). The ARRIVE guidelines 2.0: Updated guidelines for reporting animal research. PLoS Biol..

[120] Charan J., Kantharia N.D. (2013). How to calculate sample size in animal studies?. J. Pharmacol. Pharmacother..

[121] Matsubara N.K., da Silva-Santos J.E. (2024). The dual cardiovascular effect of centrally administered clonidine: A comparative study between pentobarbital- and ketamine/xylazine-anesthetized rats. Future Pharmacol..

[122] Kaltuş Z., Harmanci N., Eroğlu E., Özatik O., Yildirim E. (2025). The effect of histamine H4 Receptor Antagonist JNJ7777120 on Experimental Ulcer Models in Rats. Clin. Exp. Health Sci..

[123] Izzettin F.V., Sancar M., Okuyan B., Apikoglu-Rabus S., Cevikbas U. (2012). Comparison of the protective effects of various antiulcer agents alone or in combination on indomethacin-induced gastric ulcers in rats. Exp. Toxicol. Pathol..

[124] Coruzzi G., Adami M., Guaita E., de Esch I.J.P., Leurs R. (2007). Antiinflammatory and antinociceptive effects of the selective histamine H4-receptor antagonists JNJ7777120 and VUF6002 in a rat model of carrageenan-induced acute inflammation. Eur. J. Pharmacol..

[125] Yildiz G., Yildiz Y., Ulutas P.A., Yaylali A., Ural M. (2015). Resveratrol pretreatment ameliorates TNBS colitis in rats. Recent Pat. Endocr. Metab. Immune Drug Discov..

[126] Wellington D., Mikaelian I., Singer L. (2013). Comparison of ketamine-xylazine and ketamine-dexmedetomidine anesthesia and intraperitoneal tolerance in rats. J. Am. Assoc. Lab. Anim. Sci..

[127] Parra R.S., Lopes A.H., Carreira E.U., Feitosa M.R., Cunha F.Q., Garcia S.B., Cunha T.M., da Rocha J.J., Féres O. (2015). Hyperbaric oxygen therapy ameliorates TNBS-induced acute distal colitis in rats. Med. Gas Res..

[128] Yeni Y., Cakir Z., Hacimuftuoglu A., Taghizadehghalehjoughi A., Okkay U., Genc S., Yildirim S., Saglam Y.S., Calina D., Tsatsakis A. (2022). A selective histamine H4 receptor antagonist, JNJ7777120, role on glutamate transporter activity in chronic depression. J. Pers. Med..

[129] Oyanagui Y. (1984). Reevaluation of assay methods and establishment of kit for superoxide dismutase activity. Anal. Biochem..

[130] Aebi H. (1984). Catalase in vitro. Methods Enzymol..

[131] Habig W.H., Jakoby W.B. (1981). Assays for differentiation of glutathione S-transferases. Methods Enzymol..

[132] Kobayashi J., Sasaki D., Kondo A. (2018). A procedure for precise determination of glutathione produced by Saccharomyces cerevisiae. Bio-protocol.

[133] Ohkawa H., Ohishi N., Yagi K. (1979). Assay for lipid peroxides in animal tissues by thiobarbituric acid reaction. Anal. Biochem..

[134] Erel O. (2004). A novel automated method to measure total antioxidant response against potent free radical reactions. Clin. Biochem..

[135] Erel O. (2005). A new automated colorimetric method for measuring total oxidant status. Clin. Biochem..

[136] van Asperen K. (1962). A study of housefly esterases by means of a sensitive colorimetric method. J. Insect Physiol..

[137] Bradford M. (1976). A rapid and sensitive method for the quantification of microgram quantities of protein utilizing the principle of protein-dye binding. Anal. Biochem..

